# Diagnostic and prognostic value of presepsin and procalcitonin in non-infectious organ failure, sepsis, and septic shock: a prospective observational study according to the Sepsis-3 definitions

**DOI:** 10.1186/s12879-021-07012-8

**Published:** 2022-01-04

**Authors:** Sukyo Lee, Juhyun Song, Dae Won Park, Hyeri Seok, Sejoong Ahn, Jooyeong Kim, Jonghak Park, Han-jin Cho, Sungwoo Moon

**Affiliations:** 1 Department of Emergency Medicine, Korea University Ansan Hospital, Ansan, Republic of Korea; 2 Division of Infectious Diseases, Department of Internal Medicine, Korea University Ansan Hospital, Ansan, Republic of Korea

**Keywords:** Presepsin, Procalcitonin, Organ failure, Sepsis, Septic shock, Mortality

## Abstract

**Background:**

We investigated the diagnostic and prognostic value of presepsin among patients with organ failure, including sepsis, in accordance with the Third International Consensus Definitions for Sepsis and Septic Shock (Sepsis-3).

**Methods:**

This prospective observational study included 420 patients divided into three groups: non-infectious organ failure (n = 142), sepsis (n = 141), and septic shock (n = 137). Optimal cut-off values of presepsin to discriminate between the three groups were evaluated using receiver operating characteristic curve analysis. We determined the optimal cut-off value of presepsin levels to predict mortality associated with sepsis and performed Kaplan–Meier survival curve analysis according to the cut-off value. Cox proportional hazards model was performed to determine the risk factors for 30-day mortality.

**Results:**

Presepsin levels were significantly higher in sepsis than in non-infectious organ failure cases (*p* < 0.001) and significantly higher in patients with septic shock than in those with sepsis (*p* = 0.002). The optimal cut-off value of the presepsin level to discriminate between sepsis and non-infectious organ failure was 582 pg/mL (p < 0.001) and between sepsis and septic shock was 1285 pg/mL (*p* < 0.001). The optimal cut-off value of the presepsin level for predicting the 30-day mortality was 821 pg/mL (*p* = 0.005) for patients with sepsis. Patients with higher presepsin levels (≥ 821 pg/mL) had significantly higher mortality rates than those with lower presepsin levels (< 821 pg/mL) (log-rank test; *p* = 0.004). In the multivariate Cox proportional hazards model, presepsin could predict the 30-day mortality in sepsis cases (hazard ratio, 1.003; 95% confidence interval 1.001–1.005; *p* = 0.042).

**Conclusions:**

Presepsin levels could effectively differentiate sepsis from non-infectious organ failure and could help clinicians identify patients with sepsis with poor prognosis. Presepsin was an independent risk factor for 30-day mortality among patients with sepsis and septic shock.

## Background

Sepsis is a life-threatening organ dysfunction caused by a dysregulated host response to infection [1]. Despite advances in management, sepsis is the leading cause of mortality in critically ill patients [2, 3]. According to the Surviving Sepsis Campaign (SSC) guidelines, early diagnosis of sepsis and therapeutic interventions are essential to improve survival outcomes [2, 4, 5]. Though the Third International Consensus Definitions for Sepsis and Septic Shock (Sepsis-3) has been published [1, 6], no single gold standard diagnostic method for sepsis has been identified. Blood cultures can determine the presence of bacteremia, but it usually takes a few days to obtain microbiological results and yields false negative results in many cases. Thus, various novel biomarkers to determine the presence of infection have been evaluated, and some markers, such as C-reactive protein (CRP) and procalcitonin (PCT), are widely used in clinical settings [7, 8]. PCT is the inactive propeptide of calcitonin, which is released by C cells of the thyroid gland, hepatocyte, and peripheral monocytes. Though PCT exhibits higher specificity for bacterial infection than CRP and other traditional markers do, its level may also be elevated in conditions without infection [7, 9]. PCT also appears to have a limited ability to predict mortality associated with sepsis [10].

The soluble cluster of differentiation 14 subtype, presepsin, is a novel and promising biomarker identified in 2005 [8]. Presepsin is a soluble N-terminal fragment of the cluster of differentiation (CD) marker protein CD14. CD14 is a free fragment of glycoprotein expressed on monocyte and macrophage [8, 10]. It is a receptor of lipopolysaccharide–lipopolysaccharide-binding protein (LPS-LBP) complexes, transducing the endotoxin signal from bacterial infection through the Toll-like receptor-4. Its soluble form, soluble CD14 (sSD14), is produced from cell secretion or when membrane-bound, CD14 (mCD14) detaches from cells such as phagocytes [8, 11]. The N-terminal fragments of 13 kDa consist of sCD14 subtype (sCD14-ST; presepsin) are related to mediating the immune response to LPS [10, 11]. PCT level increases in 8–24 h and reaches the peak later than 24 h after infection, while presepsin level typically increases within 2 h and reaches the peak in 3 h [11].

Presepsin was reported to have diagnostic and prognostic abilities in patients with sepsis in some studies performed according to the previous Sepsis-2 definitions [11–13]. A systematic review and meta-analysis showed that the diagnostic accuracy of presepsin in detecting infection was similar to that of PCT, and both biomarkers were useful for the early diagnosis of sepsis [14, 15]. A recent study using Sepsis-3 reported that presepsin and PCT were superior to CRP and lactate in discriminating sepsis and septic shock from systemic inflammatory response syndrome (SIRS) without infection [16]. Another study using the Sepsis-3 definition also showed that presepsin could effectively discriminate sepsis without shock from non-sepsis with an increase in sepsis-related organ failure assessment (SOFA) score of ≥ 2 [17]. However, to the best of our knowledge, there has been no study on the diagnostic and prognostic value of presepsin in patients with organ failure in the emergency department (ED) according to the latest Sepsis-3 definitions. We hypothesized that presepsin could have diagnostic and prognostic value in organ failure, including sepsis, diagnosed using Sepsis-3. Therefore, we aimed to investigate the diagnostic value of presepsin levels in patients with non-infectious organ failure, sepsis, and septic shock, as well as the prognostic value of presepsin levels in patients with sepsis and septic shock. Although both presepsin and PCT commonly increase in the response of a host to microbial infection, they have differences in the mechanism of secretion and peak time after infection. We compared the diagnostic and prognostic performance of presepsin and PCT to provide clinicians with novel evidence for sepsis care in ED.

## Methods

### Study design and setting

This single-center prospective observational study was performed at the ED of the Korea University Ansan Hospital, Korea. Our institution is a 910-bed (44 intensive care unit) tertiary care teaching hospital with approximately 50,000 annual patient visits to the ED. This study was conducted in accordance with the Declaration of Helsinki (2013; Seventh revision, 64th Meeting, Fortaleza) and was approved by the Institutional Review Board (IRB) of Korea University Ansan Hospital (IRB no. 2020AS0031). Written informed consent was obtained from all patients or their legal representatives.

### Study population

From July 2019 to August 2020, adults (aged ≥ 18 years) who had a positive SOFA (qSOFA) score upon ED presentation were screened for participation. This scoring system used three criteria: low blood pressure (systolic blood pressure ≤ 100 mmHg), high respiratory rate (≥ 22 breaths/min), and altered mental status (Glasgow coma score < 15). One point was assigned for each criterion, with a final score ranging from 0 to 3 points. A positive qSOFA score was defined as a qSOFA score of ≥ 2. In the present study, another inclusion criterion was an increase in the SOFA score by ≥ 2 points in the ED, irrespective of the current infection. Since September 2017, our institution has been using the Intelligent Sepsis Management System (i-SMS), a qSOFA alert system, which helps ED clinicians promptly identify sepsis and manage sepsis according to the SSC 2016 guidelines [4, 5]. The system automatically enrolled patients who had a positive qSOFA score upon ED arrival and assisted in the decision-making process for sepsis management. If patients had baseline SOFA scores, we used the standard of an increase in SOFA score of at least 2. If patients had no previous SOFA score, two infectious disease (ID) experts independently reviewed the medical records containing laboratory data and determined the change in the SOFA score. Exclusion criteria were refusal to provide consent, an increase in SOFA scores < 2, ED visit for trauma care, and unknown outcomes. Therefore, all enrolled patients had a qSOFA score ≥ 2 or an increase in the SOFA score by ≥ 2 points. Two ID experts and an emergency attending physician independently classified eligible patients into the following three groups based on the presence of infection and sepsis severity: non-infectious organ failure, sepsis, and septic shock. All three physicians were blinded to the levels of presepsin and procalcitonin. Non-infectious organ failure group is a control group, while septic group is an experimental group, which consists of two subgroups-sepsis and septic shock. The light kappa value for the three raters (i.e., the average kappa value across all rater pairs) was 0.928. After careful discussion of a few discrepancies, the three raters agreed on the classification.

### Data collection

Data on vital signs, laboratory tests, biomarker levels (presepsin and PCT), blood gas analysis, Glasgow coma scale scores, blood culture, and demography (age, sex, body weight, and prior medical history) in the ED were collected and documented by assistant researchers. We completed missing clinical data using multiple imputation. Patients were followed up for 90 days after ED presentation. If they were discharged or transferred to other institutions earlier than 90 days after ED presentation, we collected data through telephone conversations with patients, their legal representatives, or their physicians.

### Definitions

According to Sepsis-3 definitions, sepsis is a life-threatening organ dysfunction caused by a dysregulated host response to infection [1]. Septic shock is defined as a subset of sepsis in which profound circulatory, cellular, and metabolic abnormalities pose a greater risk of mortality than sepsis alone [1, 6]. Sepsis-3 recommends the use of the qSOFA score to identify patients with poor prognosis outside the intensive care unit (ICU). The diagnostic criteria for sepsis include an increase in the SOFA score by ≥ 2 points owing to current infection. The criteria for septic shock include the requirement for a vasopressor to maintain a mean arterial pressure of 65 mmHg and serum lactate level > 2 mmol/L despite adequate fluid resuscitation. Finally, the criteria for “non-infectious organ failure” included a positive qSOFA score and an increase in SOFA score by ≥ 2 points without current infection. Two ID experts independently reviewed the medical records and laboratory results to determine the presence of a current infection.

### Assays

We sampled blood for presepsin and PCT testing from a peripheral vein within 6 h of ED presentation. We put blood sample in the ethylenediamine tetraacetic acid (EDTA) tubes, and deposited the sample in the Biobank of our institution. Plasma was separated in EDTA tube. The aliquots were frozen at − 80 °C until analysis in the Biobank. In accordance with the suggestion of the previous studies and commercial kits, we used plasma for presepsin and procalcitonin measurement. According to the package insert of the PATHFAST Presepsin kit, plasma samples are stable for only 3 days at 2 to 8 °C and 9 months at − 20 °C or lower. If the sample storage period was more than 9 months, the test result may not be reliable and a bias may be introduced. Procalcitonin is stable at room temperature for only 24 h and stable frozen for 3 months. Therefore, all the measurements for both biomarkers were performed within 3 months from sample collection in the emergency department. Blood samples were collected from the Biobank of our institution. Therefore, the biospecimens and data used for this study were provided by the Biobank of Korea University Ansan Hospital. Plasma presepsin levels were measured using an automated chemiluminescent enzyme immunoassay (PATHFAST system, LSI Medience Corporation, Tokyo, Japan). This novel system, based on the chemiluminescent enzyme immunoassay principle, was developed to analyze blood samples, providing results within 17 min [18]. During incubation of the sample with alkaline phosphatase (ALP)-labeled anti-presepsin polyclonal antibodies and anti-presepsin monoclonal antibody-coated magnetic particles, presepsin binds to anti-presepsin antibodies, assembling an immunocomplex with the ALP-labeled antibodies and mouse monoclonal antibody-coated magnetic particles. The manufacturer-claimed assay range of presepsin was 20–20,000 pg/mL. Plasma presepsin concentrations were measured after the enrolled patients were discharged from the ED. Therefore, the assay results were unavailable to ED physicians and could not influence the management and disposition of the patients. PCT levels were measured using the Elecsys BRAHMS procalcitonin automated electrochemiluminescence assay (BRAHMS, Henningsdorf, Germany) on the Roche Cobas e-System (Roche Diagnostics, Basel, Switzerland). The manufacturer-claimed assay range of PCT was 0.02–100 ng/mL.

### Outcomes

The primary outcome was the 30-day mortality, and the secondary outcome was the 90-day mortality. We excluded patients who were lost to follow-up from the 30-day-and 90-day analyses.

### Statistical analysis

Based on the results of a previous study, we expected the 30-day all-cause mortality to be 35% among patients with sepsis. The study showed that the area under the receiver operating characteristic (ROC) curve values used to discriminate sepsis from non-sepsis were 0.88 for presepsin and 0.81 for PCT. We hypothesized that similar area under the curve (AUC) would be observed in the present study. Assuming 90% power with 2-sided alpha levels of 0.05, our study required 360 patients (128 patients with non-infectious organ failure and 232 patients with sepsis including shock). Statistical analyses were performed using MedCalc for Windows (version 19.1.6; MedCalc Software, Mariakerke, Belgium) and SPSS (version 23.0; IBM, Armonk, NY, USA). A statistician from our institution oversaw all analyses during the study period. Statistical significance was set at *p* < 0.05. To compare clinical characteristics and outcomes (7-, 14-, 30-, and 90-day mortality) between the three groups, continuous variables, presented as median and interquartile range (IQR), were compared using the Kruskal–Wallis test. Data were tested for normality using the Kolmogorov–Smirnov and Shapiro–Wilk tests. Categorical variables, presented as numbers and percentages, were compared using the Chi-square test or Fisher’s exact test. Pairwise comparisons were performed separately for each pair of the three groups. The Bonferroni method was used to adjust the *p*-values in the post-hoc analysis. To compare baseline characteristics between survivors and non-survivors among patients with sepsis and septic shock, continuous variables, presented as the median and IQR, were compared using Student’s t-test or the Mann–Whitney test according to the data distribution. Categorical variables, presented as numbers and percentages, were compared using the Chi-square test or Fisher’s exact test. ROC curve analyses were performed for individual biomarkers, and their diagnostic values for sepsis and septic shock were compared. The discriminating abilities of the tested biomarkers are presented as the AUC with a 95% confidence interval (CI). The optimal cut-off value was identified for each ROC curve using the Youden index (maximum sum of sensitivity and specificity). ROC curve analysis was performed for presepsin to predict the 30-day mortality. The optimal cut-off value of the presepsin level for predicting the 30-day mortality was set using the Youden index. Kaplan–Meier survival curve analysis and log-rank tests were performed according to the cut-off values of presepsin levels. Univariate and multivariate Cox hazard model analyses were performed to evaluate the risk factors for the 30-day mortality among patients with sepsis.

## Results

### Baseline characteristics

**Fig. 1:**
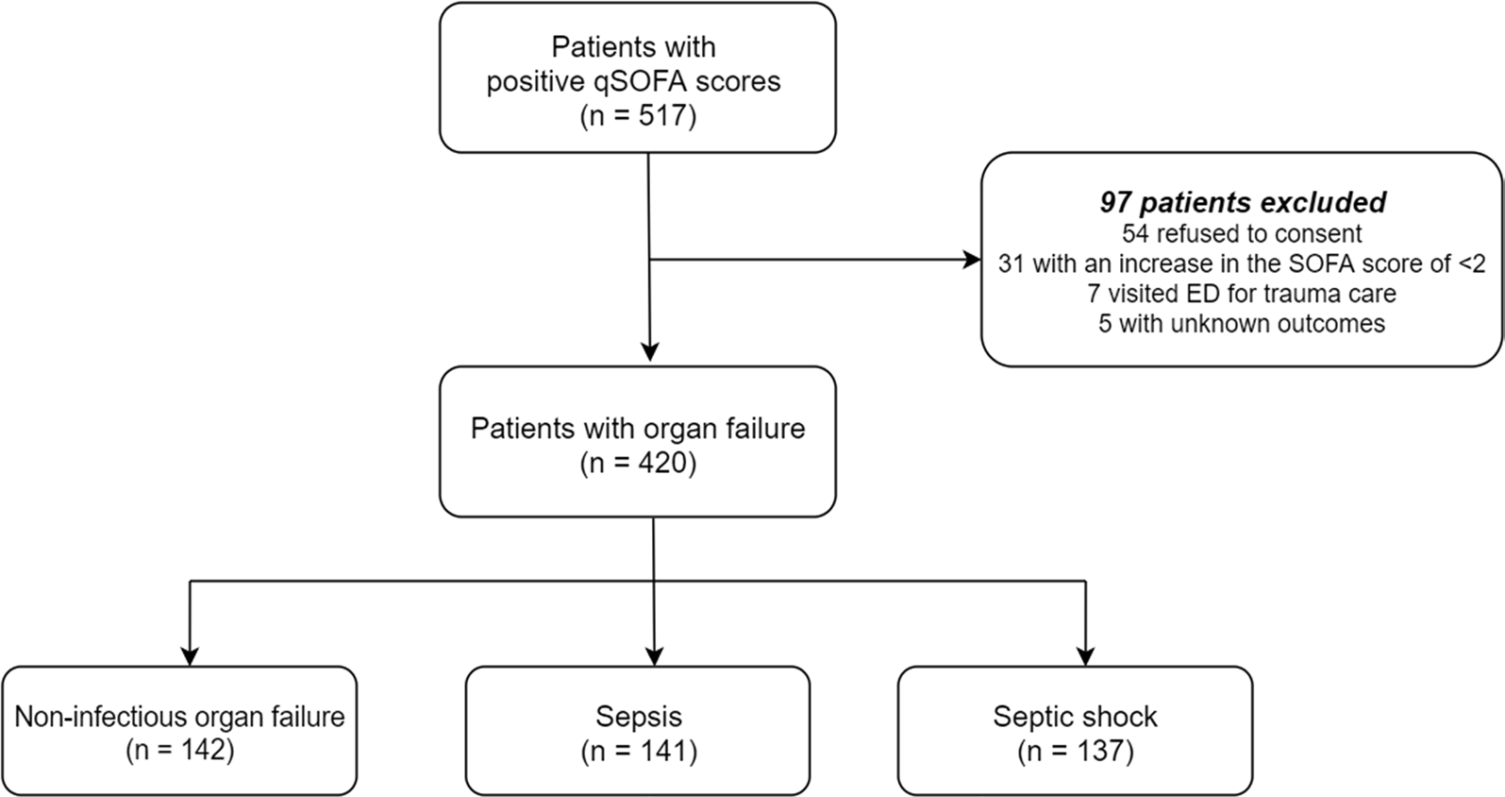
Flowchart of the study population. *qSOFA* quick sequential organ failure assessment, *SOFA* sequential organ failure assessment, *ED* emergency department

During the study period, 517 patients with positive qSOFA scores upon presentation to the ED were screened using the i-SMS (Fig. 1). Among them, 97 patients were excluded owing to refusal to participate (n = 54), increase in SOFA scores of < 2 (n = 31), admission for trauma care (n = 7), or unknown outcomes (loss to follow-up) (n = 5). The final study population consisted of 420 patients. Of them, 142 had non-infectious organ failure, 141 had sepsis, and 137 had septic shock. A flowchart of the study population is shown in Fig. 1. Baseline characteristics of the study population are shown in Table 1. Patients with sepsis and septic shock were older than those with non-infectious organ failure. Sex and the Charlson comorbidity index did not differ between the three groups. Acute Physiology and Chronic Health Evaluation (APACHE) II, SOFA, National Early Warning (NEWS), and Modified Early Warning (MEWS) scores were significantly higher for sepsis and septic shock patients than for non-infectious organ failure patients. The 7-, 14-, 30-, and 90-day mortality rates were higher among patients with septic shock than among other groups. Table 2 shows the principal clinical diagnoses of patients with non-infectious organ failure according to the affected organ systems: 52, central nervous system disorders; 41, cardiovascular disorders; 21, respiratory disorders; 19, hepatobiliary disorders; 14, renal disorders; and 7, coagulation disorders. The most common diagnoses were hypovolemic shock, metabolic encephalopathy, cerebral hemorrhage, heart failure, chronic obstructive pulmonary disease, asthma, seizure, and liver cirrhosis (Table 2).

**Table 1:** Baseline characteristics of the study population

Variables	Non-infectious organ failure (n = 142)	Sepsis (n = 141)	Septic shock (n = 137)	*p* value
Age, median (IQR)	66 (51–80)	76 (67–83)	77 (62–83)	< 0.001
Male, n (%)	85 (56)	85 (62)	74 (57)	0.519
Charlson comorbidity index	4 (3–5)	3 (3–5)	5 (4–6)	0.182
Underlying diseases, n (%)				
Diabetes mellitus	37 (26)	54 (38)	51 (37)	0.055
Hypertension	60 (42)	78 (55)	66 (48)	0.089
Malignancy	23 (16)	23 (16)	19 (14)	0.818
Chronic liver disease	9 (6)	8 (6)	11 (8)	0.720
Chronic kidney disease	7 (5)	16 (11)	11 (8)	0.141
Cardiovascular disease	10 (7)	15 (11)	13 (10)	0.560
Site of infection, n (%)				
Respiratory		84 (60)	81 (59)	0.713
Genitourinary		35 (25)	33 (24)	0.367
Gastrointestinal		14 (10)	13 (10)	0.386
Others		13 (9)	15 (11)	0.281
Positive blood culture, n (%)		60 (43)	62 (45)	0.412
APACHE II score, median (IQR)	23 (18–29)	26 (22–32)	29 (25–33)	< 0.001
SOFA score, median (IQR)	6 (3–8)	6 (5–8)	10 (8–12)	< 0.001
NEWS, median (IQR)	9 (7–11)	10 (8–12)	11 (9–14)	< 0.001
MEWS, median (IQR)	5 (4–7)	6 (5–7)	7 (6–9)	< 0.001
WBC (× 10^9^/L), median (IQR)	11.30 (8.17–14.63)	11.94 (8.24–17.06)	11.22 (6.68–20.04)	0.343
CRP (mg/dL), median (IQR)	0.53 (0.13–2.42)	7.50 (3.33–16.66)	10.07 (3.99–20.70)	< 0.001
Procalcitonin (ng/mL), median (IQR)	0.10 (0.05–0.25)	0.98 (0.35–4.25)	4.22 (0.88–21.02)	< 0.001
Presepsin (pg/mL), median (IQR)	286 (170–417)	792 (450–1273)	1287 (589–2366)	< 0.001
Lactate (mmol/L), median (IQR)	2.0 (1.4–4.6)	2.2 (1.5–4.9)	4.4 (2.4–8.1)	< 0.001
Creatinine (mg/dL), median (IQR)	1.3 (0.9–2.1)	1.3 (0.9–2.5)	1.7 (1.1–2.6)	< 0.001
Bilirubin (mg/dL), median (IQR)	0.6 (0.4–1.1)	0.7 (0.4–1.3)	0.7 (0.4–1.2)	0.003
Platelet (× 1000/μL), median (IQR)	200 (139–286)	206 (142–301)	185 (102–258)	0.002
Length of hospital stay (days), median (IQR)	8 (3–15)	8 (3–14)	11 (4–17)	0.243
Length of ICU stay (days), median (IQR)	4 (2–5)	4 (2–6)	5 (2–7)	0.218
7-day mortality	11 (7.2)	11 (8.0)	33 (25.2)	< 0.001
14-day mortality	16 (10.5)	19 (13.9)	40 (30.5)	< 0.001
30-day mortality	20 (13.2)	22 (16.1)	47 (35.9)	< 0.001
90-day mortality	21 (13.8)	33 (24.1)	52 (39.7)	< 0.001

*IQR* interquartile range, *APACHE* Acute Physiology and Chronic Health Evaluation, *SOFA* sepsis-related organ failure assessment, *NEWS* National Early Warning Score, *MEWS* Modified Early Warning Score, *WBC* white blood cell, *CRP* C-reactive protein, *ICU* intensive care unit

**Table 2:** Principal diagnoses of non-infectious organ failure patients (n = 142) according to affected organ systems

Organs	Main clinical diagnoses	n (%)
Central nervous system (n = 52)	Cerebral hemorrhage (ICH, IVH, SAH, and SDH)	12 (8.5)
	Cerebral infarction	5 (3.5)
	Seizure	11 (7.7)
	Hypoglycemia	4 (2.8)
	Metabolic encephalopathy	13 (9.2)
	Heat stroke	2 (1.4)
	Others	5 (3.5)
Cardiovascular (n = 41)	Heart failure	12 (8.5)
	Pulmonary embolism	5 (3.5)
	Hypovolemic (hemorrhagic) shock	17 (12.0)
	Aortic dissection	4 (2.8)
	Others	3 (2.1)
Respiratory (n = 21)	COPD or asthma	12 (8.5)
	Malignancy in respiratory system	4 (2.8)
	Airway obstruction	3 (2.1)
	Others	2 (1.4)
Hepatobiliary (n = 19)	Liver cirrhosis aggravation	11 (7.7)
	Hepatobiliary malignancy	5 (3.5)
	Others	3 (2.1)
Renal (n = 14)	Acute kidney injury	9 (6.3)
	Under dialysis in pre-existing CKD	5 (3.5)
Coagulation (n = 7)	Hematologic malignancy	4 (2.8)
	Thrombocytopenia	3 (2.1)

*ICH* intracerebral hemorrhage, *IVH* intraventricular hemorrhage, *SAH* subarachnoid hemorrhage, *SDH* subdural hematoma, *COPD* chronic obstructive pulmonary disease, *CKD* chronic kidney disease

### Presepsin, PCT, and CRP measurement

**Fig. 2:**
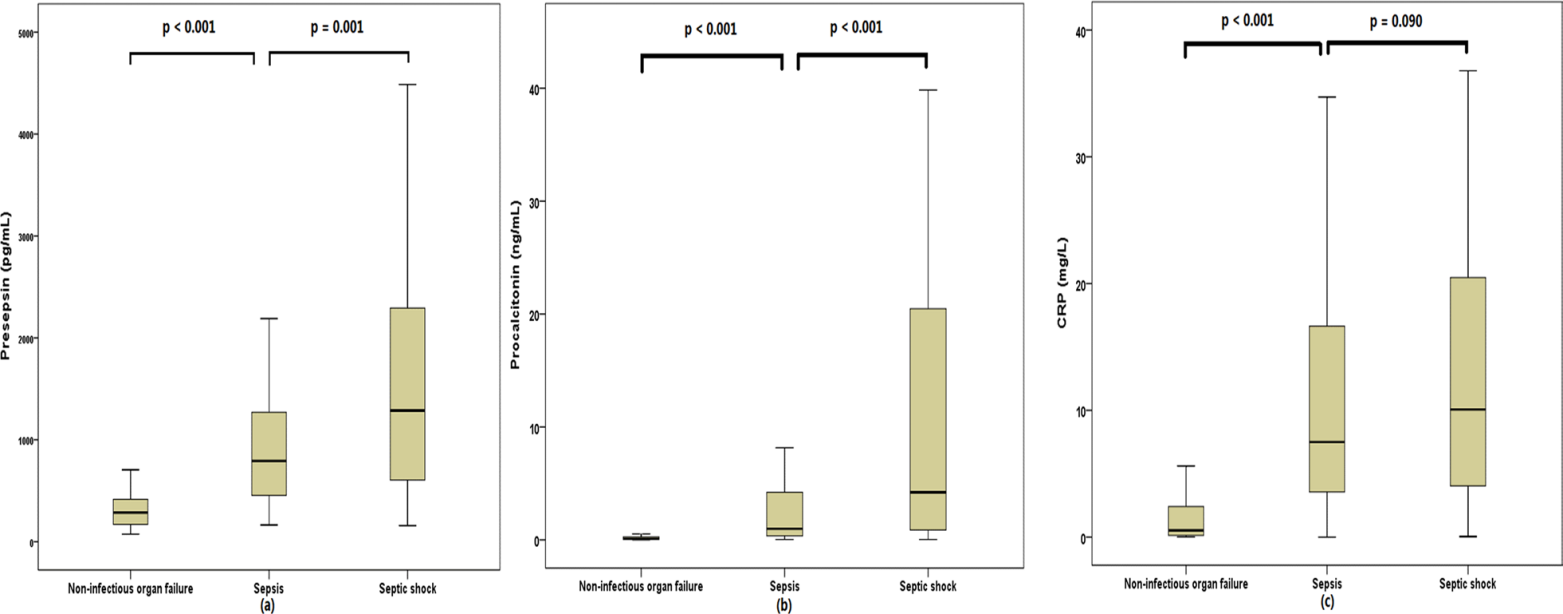
Presepsin (**a**), procalcitonin (**b**), and CRP (**c**) levels among ED patients with organ failure. *CRP* C-reactive protein, *ED* emergency department

A comparison of presepsin, PCT, and CRP levels among patients with organ failure is shown in Fig. 2 and Table 1. Presepsin, PCT, and CRP levels were significantly higher in patients with sepsis or septic shock than in those with non-infectious organ failure. Presepsin and PCT levels were significantly higher in patients with septic shock than in those with sepsis. In contrast, we observed no significant differences in CRP levels between the sepsis and septic shock groups (Fig. 2).

### Correlations with other biomarkers and severity scores

Presepsin levels positively correlated with creatinine (rho = 0.588, *p* < 0.001), PCT (rho = 0.535, *p* < 0.001), CRP (rho = 0.386, *p* < 0.001), bilirubin (rho = 0.325, *p* < 0.001), lactate (rho = 0.230, *p* < 0.001) levels and SOFA (rho = 0.504, *p* < 0.001) and APACHE II scores (rho = 0.437, *p* < 0.001). Presepsin levels negatively correlated with platelet counts (rho = − 0.254, *p* < 0.001).

### Diagnostic value of presepsin, PCT, and CRP

**Fig. 3:**
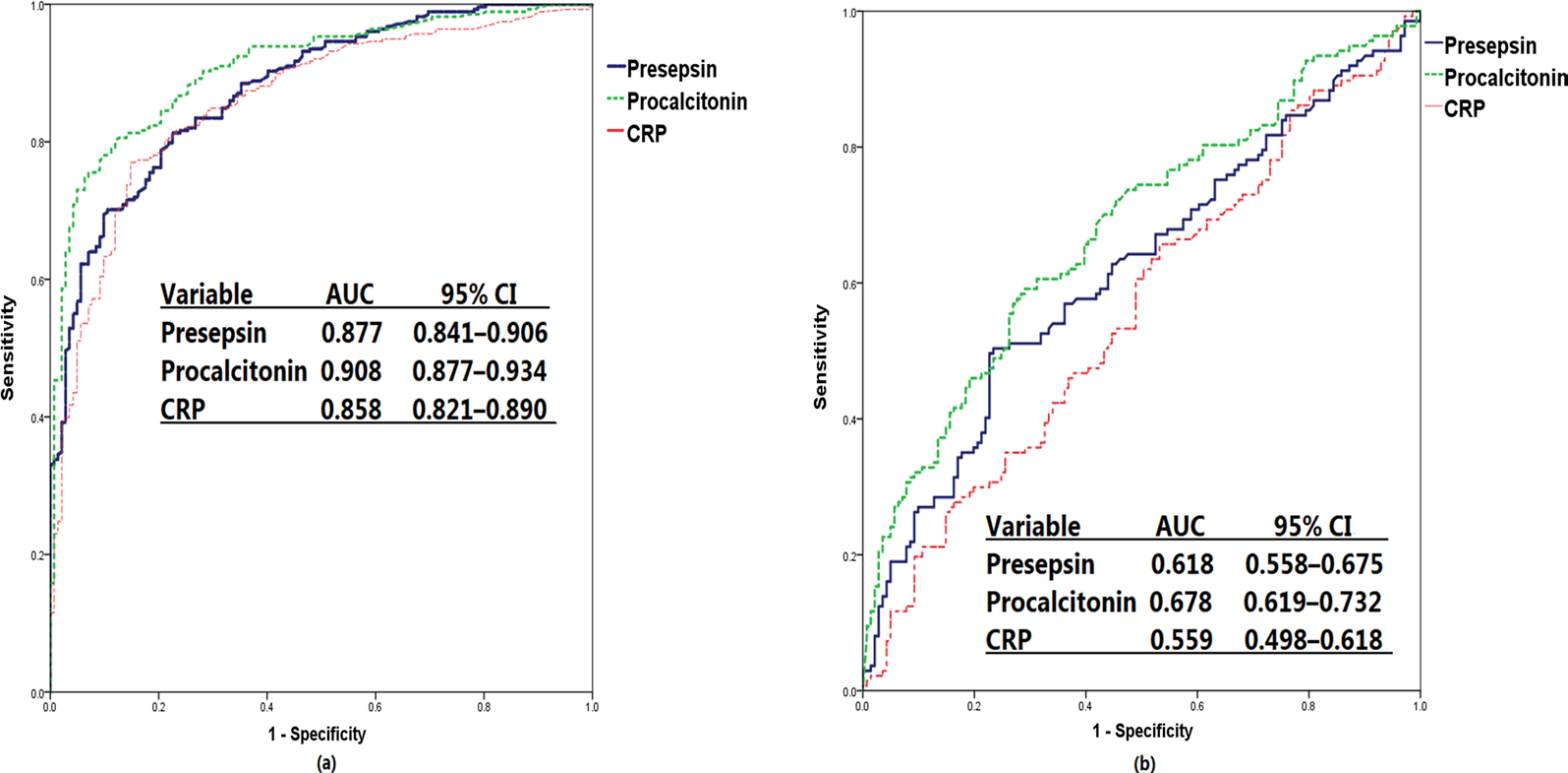
Receiver operating characteristic curves of presepsin, procalcitonin, and CRP levels. Discrimination of sepsis (including shock) from non-infectious organ failure (**a**) and septic shock from sepsis (**b**). *CRP* C-reactive protein

ROC curve analyses to discriminate between the three groups are shown in Fig. 3 and Table 3. The optimal cut-off value of the presepsin level to discriminate between sepsis and non-infectious organ failure was 582 pg/mL (sensitivity, 70.1%; specificity, 89.4; AUC, 0.877; 95% CI 0.841–0.906; *p* < 0.001). The optimal cut-off value of the presepsin level to discriminate between sepsis and septic shock was 1285 pg/mL (sensitivity, 50.4%; specificity, 76.6%; AUC, 0.618; 95% CI 0.558–0.675; *p* < 0.001). The optimal cut-off value of the PCT level to discriminate between sepsis and non-infectious organ failure was 0.51 ng/mL (sensitivity, 75.5%; specificity, 93.0%; AUC, 0.908; 95% CI 0.877–0.934; *p* < 0.001). The optimal cut-off value of the PCT level to discriminate between sepsis and septic shock was 2.81 ng/mL (sensitivity, 59.1%; specificity, 70.9%; AUC, 0.678%; 95% CI 0.619–0.732; *p* < 0.001). The optimal cut-off value of the CRP level to discriminate between sepsis and non-infectious organ failure was 3.53 mg/L (sensitivity, 77.0%; specificity, 85.2%; AUC, 0.858; 95% CI 0.821–0.890; *p* < 0.001). The optimal cut-off value of the CRP level to discriminate between sepsis and septic shock was 6.62 mg/L (sensitivity, 65.7%; specificity, 46.8%; AUC, 0.559; 95% CI 0.498–0.618; *p* = 0.088).

**Table 3:** Comparisons of discriminating abilities of tested biomarkers presented as areas under the curve (95% CI)

Tested biomarker	AUC (95% CI)	*p* value	Cut-off value	Sensitivity, (%)	Specificity, (%)
Presepsin					
Sepsis vs. non-infectious organ failure	0.877 (0.841–0.906)	< 0.001	582 (pg/mL)	70.1	89.4
Septic shock vs. sepsis	0.618 (0.558–0.675)	< 0.001	1285 (pg/mL)	50.4	76.6
Procalcitonin					
Sepsis vs. non-infectious organ failure	0.908 (0.877–0.934)	< 0.001	0.51 (ng/mL)	75.5	93.0
Septic shock vs. sepsis	0.678 (0.619–0.732)	< 0.001	2.81 (ng/mL)	59.1	70.9
CRP					
Sepsis vs. non-infectious organ failure	0.858 (0.821–0.890)	< 0.001	3.53 (mg/L)	77.0	85.2
Septic shock vs. sepsis	0.559 (0.498–0.618)	0.088	6.62 (mg/L)	65.7	46.8

*AUC* area under the curve, *CRP* C-reactive protein

### Prognostic value of presepsin

**Fig. 4:**
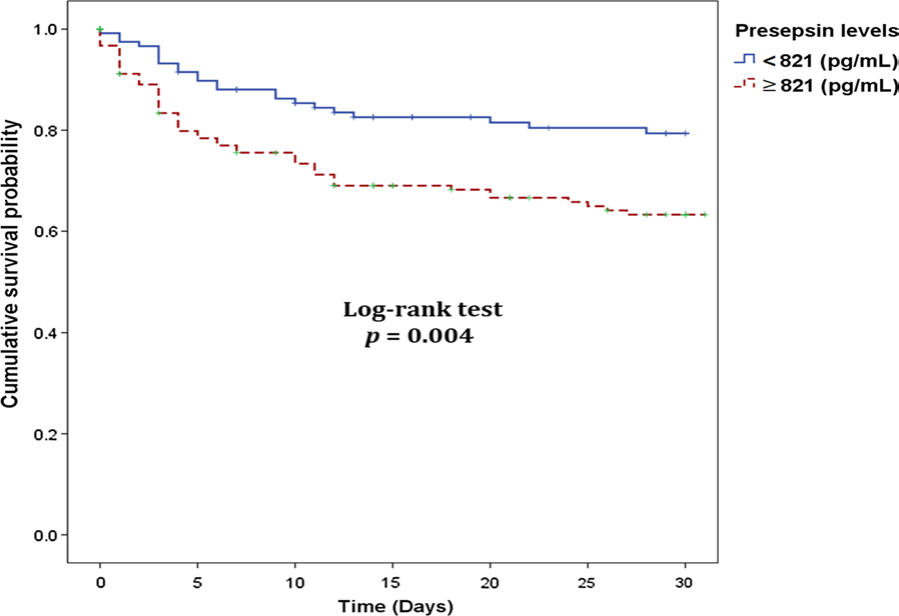
Prediction of 30-day mortality in patients with sepsis and septic shock. Kaplan–Meier survival curve analysis and log-rank test according to the optimal cut-off of presepsin for predicting 30-day mortality in patients with sepsis and septic shock

The 30-day mortality rate was 27% (74/278) among patients with sepsis and septic shock (Table 4). We compared clinical variables between 30-day survivors and non-survivors among patients with sepsis (non-infectious organ failure excluded). Survivors and non-survivors did not differ in age, sex, Charlson comorbidity index, sites of infection, and CRP or PCT levels. APACHE II, SOFA, NEWS, and MEWS scores were significantly higher for non-survivors than for survivors. Presepsin levels were significantly higher in non-survivors than in survivors (1142 [650–2039] ng/mL vs. 815 [460–1678] ng/mL; *p* < 0.001). Lactate levels were significantly higher in non-survivors than in survivors (6.0 [2.9–9.9] mmol/L vs. 2.6 [1.6–5.2] mmol/L; *p* < 0.001). The optimal cut-off value of the presepsin level for predicting 30-day mortality was 821 pg/mL (sensitivity, 68.9%; specificity, 50.5%; AUC, 0.605; 95% CI 0.545–0.663; *p* = 0.005) in patients with sepsis and septic shock. The 30-day mortality rates were 18.4% (23/125) among patients with lower presepsin levels (< 821 pg/mL) and 33.3% (51/153) among patients with higher presepsin levels (≥ 821 pg/mL). Kaplan–Meier survival curve analysis showed that patients with higher presepsin levels had significantly higher mortality than patients with lower presepsin levels (log-rank test; *p* = 0.004) (Fig. 4). Univariate and multivariate Cox proportional hazards model analyses of the 30-day mortality are shown in Table 5. Univariate analysis determined that the significant risk factors for the 30-day mortality were SOFA and APACHE II scores, septic shock, lactate level, presepsin level, platelet count, and white blood cell (WBC) count. The significant risk factors identified in multivariate analysis were presepsin level (HR, 1.003; 95% CI 1.001–1.005; *p* = 0.042), SOFA score (hazards ratio [HR], 1.264; 95% CI 1.167–1.369; *p* < 0.001), and lactate level (HR, 1.108; 95% CI 1.070–1.147; *p* < 0.001).

**Table 4:** Clinical variables comparison between 30-day survivors and non-survivors among sepsis cases (non-infectious organ failure excluded)

Variables	All septic patients (n = 278)	Survivors (n = 204)	Non-survivors (n = 74)	*p* value
Age, median (IQR)	77 (64–84)	77 (64–83)	78 (65–85)	0.210
Male, n (%)	162 (58)	120 (59)	42 (57)	0.757
Charlson comorbidity index	4 (3–5)	3 (3–5)	5 (4–6)	0.157
Site of infection, n (%)				
Respiratory	165 (59)	119 (58)	46 (62)	0.658
Genitourinary	68 (24)	48 (24)	20 (27)	0.412
Gastrointestinal	27 (10)	20 (10)	7 (9)	0.348
Others	28 (10)	21 (10)	7 (9)	0.316
Septic shock, n (%)		85 (42)	52 (70)	< 0.001
APACHE II score, median (IQR)	28 (24–33)	27 (22–31)	31 (26–37)	< 0.001
SOFA score, median (IQR)	9 (6–11)	8 (6–10)	11 (9–12)	< 0.001
NEWS, median (IQR)	11 (9–13)	10 (9–12)	12 (10–14)	0.002
MEWS, median (IQR)	6 (5–7)	6 (5–7)	6 (5–9)	0.043
WBC (× 10^9^/L), median (IQR)	11.68 (7.65–18.07)	11.91 (8.38–19.66)	10.76 (5.06–15.75)	0.009
CRP (mg/L), median (IQR)	9.09 (3.87–17.34)	8.69 (3.57–17.07)	10.75 (4.80–19.04)	0.270
Procalcitonin (ng/mL), median (IQR)	1.74 (0.51–8.51)	1.61 (0.47–8.95)	2.06 (0.62–7.22)	0.666
Presepsin (pg/mL), median (IQR)	934 (512–1802)	815 (460–1678)	1142 (650–2039)	< 0.001
Creatinine (mg/dL), median (IQR)	1.5 (1.0–2.5)	1.4 (0.9–2.6)	1.7 (1.0–2.2)	0.627
Bilirubin (mg/dL), median (IQR)	0.7 (0.4–1.2)	0.6 (0.4–1.0)	1.0 (0.5–1.7)	< 0.001
Platelet (× 1000/μL), median (IQR)	197 (115–288)	206 (141–305)	145 (63–212)	< 0.001
Lactate (mmol/L), median (IQR)	3.1 (1.9–6.6)	2.6 (1.6–5.2)	6.0 (2.9–9.9)	< 0.001

*IQR* interquartile range, *APACHE* Acute Physiology and Chronic Health Evaluation, *SOFA* sepsis-related organ failure assessment, *NEWS* National Early Warning Score, *MEWS* Modified Early Warning Score, *WBC* white blood cell, *CRP* C-reactive protein

**Table 5:** Predictors of the 30-day mortality among patients with sepsis using the Cox proportional hazards model

	Univariate HR (95% CI)	p value	Multivariate HR (95% CI)	p value
SOFA score	1.247 (1.167–1.332)	< 0.001	1.264 (1.167–1.369)	< 0.001
APACHE II score	1.223 (1.183–1.265)	< 0.001	1.131 (0.982–1.417)	0.127
Septic shock	2.752 (1.671–4.531)	< 0.001	1.621 (0.919–2.861)	0.095
Lactate	1.113 (1.078–1.149)	< 0.001	1.108 (1.070–1.147)	< 0.001
Presepsin	1.013 (1.003–1.025)	0.018	1.003 (1.001–1.005)	0.042
Bilirubin	1.050 (0.990–1.115)	0.105	1.014 (0.927–1.109)	0.755
Platelet	0.995 (0.993–0.998)	< 0.001	0.998 (0.996–1.001)	0.216
WBC	0.973 (0.945–0.997)	0.013	0.989 (0.971–1.008)	0.083

*HR* hazard ratio, *CI* confidence interval, *SOFA* sepsis-related organ failure assessment, *APACHE* Acute Physiology and Chronic Health Evaluation, *WBC* white blood cell

## Discussion

To the best of our knowledge, this is the largest prospective observational study on both the diagnostic and prognostic value of presepsin in non-infectious organ failure, sepsis, and septic shock, in accordance with the latest Sepsis-3 definitions. Presepsin had good accuracy in discriminating sepsis from non-infectious organ failure and had fair accuracy in discriminating septic shock from sepsis. The discriminating power of presepsin was comparable with that of PCT among patients with non-infectious organ failure, sepsis, and septic shock. The prognostic value of presepsin was superior to that of PCT and CRP in patients with sepsis and septic shock. Our results showed that the optimal cut-off value to discriminate sepsis from non-infectious organ failure was 582 pg/mL (AUC, 0.877; sensitivity, 70.1%; specificity, 89.4%). Several studies have reported different performance efficacy of presepsin as an indicator of different types of infections. Optimal cut-off values (sensitivity and specificity, respectively) to discriminate sepsis from non-sepsis were 907 (70%, 83%) [19], 686 (47%, 91%) [20], 670 (70%, 81%) [21], 729 (81%, 63%) [22], 600 (86%, 72%) [23], 600 (79%, 62%) [24], 542 (77%, 76%) [25], 430 (88%, 82%) [26], and 466 (90%, 55%) pg/mL [27]. The difference in cut-off values reported by these studies may be caused by the heterogeneity of the studies in terms of clinical setting (ED vs. ICU), study design (prospective vs. retrospective), sepsis severity, comorbidities, and type of sample (plasma vs. whole blood vs. serum). However, these studies were performed according to the previous Sepsis-2 definitions. A recent study using Sepsis-3 reported that presepsin and PCT were superior to CRP and lactate in discriminating sepsis, including shock, from non-sepsis with SIRS and a SOFA score ≥ 2 [16]. The study showed that the AUC values used to discriminate sepsis from non-sepsis were 0.88 for presepsin, 0.81 for PCT, and 0.65 for CRP. The AUC value of presepsin in the study was similar to that in our study (AUC, 0.877), and the sensitivity, specificity, positive predictive value, negative predictive value, and accuracy of presepsin for diagnosing sepsis (including shock) using a cut-off value of 508 pg/mL were 87%, 86%, 93%, 76%, and 87%, respectively. The cut-off value found in a previous study (508 pg/mL) was relatively lower than that in our study (582 pg/mL). Our study is similar to a previous study in that it was performed in the ED according to the latest Sepsis-3 definitions. However, we included a much larger population and used SOFA and qSOFA as inclusion criteria instead of SIRS because it is no longer recommended as a diagnostic criterion for sepsis in the new definitions [1]. These differences might have caused the difference in cut-off values between the two studies. Another study using Sepsis-3 for a Spanish population also reported that presepsin can effectively discriminate sepsis from non-infectious SIRS [16]. However, this study using Sepsis-3 did not evaluate the prognostic value of presepsin. A previous study reported that presepsin was superior to PCT and CRP in discriminating sepsis from SIRS in acute abdominal conditions [28]. In contrast, another study showed that the diagnostic ability of presepsin was not superior to that of PCT [20], suggesting that its introduction and routine use in clinical practice were not justified. Another study also reported that presepsin did not outperform traditional biomarkers in distinguishing sepsis from SIRS and predicting mortality [29]. In fact, results reported about the diagnostic value of presepsin are controversial, probably owing to different study designs and settings. Therefore, specific decision levels are required to determine the clinical roles of presepsin in different settings of non-infectious and infectious diseases [30]. A multicenter prospective study reported that mean presepsin levels were significantly higher in non-survivors of sepsis than in survivors [24]. However, in that study, no significant correlation was observed between PCT levels and survival outcomes [24]. Similar to the previous study, our results showed that presepsin levels were significantly higher in non-survivors than in survivors. No significant differences in PCT levels were observed between the non-survivors and survivors. In our study, Kaplan–Meier survival curve analysis according to the optimal cut-off value of the presepsin level showed that the 30-day mortality was significantly higher in patients with higher presepsin levels than in their counterparts. In accordance with our study, a systematic review and meta-analysis revealed that presepsin levels on the first day had prognostic value in predicting in-hospital or the 30-day mortality in adult patients with sepsis [31]. The combination of presepsin with PCT, galectin-3, and soluble suppression of tumorigenicity-2 showed better performance in predicting mortality than the single use of presepsin for sepsis patients [10]. The study demonstrated that the combination of presepsin with other biomarkers could help clinicians predict mortality. Further studies with larger cohorts are required to determine the optimal cut-off value of the presepsin level for predicting mortality associated with sepsis. The present study had some limitations. First, though the present study included a larger sample size than that of previous studies, it was a single-center ED-based study. Thus, our results may not be applicable to other EDs or ICUs. Second, only plasma presepsin levels in the ED were measured, and follow-up changes in markers were not determined. Though a previous study reported that dynamic monitoring of presepsin could effectively predict prognosis [32, 33], other trials demonstrated that single measurements of presepsin in the ED also had valuable prognostic value in patients with sepsis [12, 24]. Third, though a previous study reported that presepsin levels were markedly elevated in patients with chronic kidney disease receiving hemodialysis [34], our study did not consider the association between kidney dysfunction and presepsin levels. Our study population included patients with kidney dysfunction. Previous studies suggested that presepsin levels are affected by not only sepsis but also kidney dysfunction. Therefore, different cut-off value, possibly higher value, might be reasonable to discriminate sepsis from non-sepsis among patients with kidney dysfunction. However, we did not evaluate the impact of kidney dysfunction on presepsin levels. Future studies should consider the impact of kidney dysfunction on presepsin levels in extracting the optimal cut-off value for discriminating sepsis from non-sepsis. Fourth, because the present study included patients with organ dysfunction enrolled in the ED, this might have resulted in selection bias. Nevertheless, we postulate that our study, based on an organ failure cohort, could reflect the clinical characteristics of patients in a real ED setting.

## Conclusions

The present study using the Sepsis-3 definitions demonstrated the diagnostic and prognostic value of presepsin levels among patients with non-infectious organ failure, sepsis, and septic shock. Its ability to discriminate sepsis, including shock, from non-infectious organ failure was good, and its prognostic ability could help clinicians prognosticate patients with sepsis. Further multicenter prospective studies with larger cohorts are warranted to determine the optimal cut-off value of presepsin levels for the diagnosis and prognosis of sepsis.

## Data Availability

The datasets supporting the conclusions of this article are available from the corresponding author on reasonable request.

## Abbreviations

ALP: Alkaline phosphatase
APACHE: Acute physiology and chronic health evaluation
AUC: Area under the curve
CRP: C-reactive protein
ED: Emergency department
ID: Infectious diseases
IQR: Interquartile range
IRB: Institutional review board
MEWS: Modified early warning score
NEWS: National early warning score
PCT: Procalcitonin
ROC: Receiver operating characteristic
SIRS: Systemic inflammatory response syndrome
SOFA: Sepsis-related organ failure assessment
SSC: Surviving sepsis campaign
WBC: White blood cell
